# Finite confinement potentials, core and shell size effects on excitonic and electron-atom properties in cylindrical core/shell/shell quantum dots

**DOI:** 10.1038/s41598-022-19118-3

**Published:** 2022-09-01

**Authors:** M. Hbibi, O. Mommadi, S. Chouef, R. Boussetta, L. Belamkadem, A. El Moussaouy, F. Falyouni, C. M. Duque, J. A. Vinasco, C. A. Duque

**Affiliations:** 1 OAPM group, Laboratory of Materials, Waves, Energy and Environment, Department of Physics, Faculty of Sciences, University Mohamed I, 60000 Oujda, Morocco; 2 The Regional Centre for the Professions of Education and Training, Oujda, 60000 Morocco; 3 Grupo de Materia Condensada-UdeA, Instituto de Física, Facultad de Ciencias Exactas y Naturales, Universidad de Antioquia UdeA, Calle 70 No. 52-21, Medellín, Colombia

**Keywords:** Electronics, photonics and device physics, Quantum physics, Electronic properties and materials, Quantum dots, Electronic properties and materials, Nanoscale materials

## Abstract

The effects of confinement potentials of the first and second materials, core size and first shell thickness on the confinement of electron, electron-donor atom, and exciton in cylindrical core/shell/shell quantum dot (CSSQD) are studied taking into account the finite confinement potential model. The confinement of charge carriers in CSSQD with two finite confinement potentials models of the barrier materials are studied. Within the effective mass and parabolic band approximation, the 3D time-independent Schrödinger equation has been resolved. To obtain the ground state quasiparticles energies, we have used the variational technique. Our results show that the donor atom and exciton binding energy, as well as the electron energy, strongly depend on the core radius, first shell thickness, confinement potentials of the barrier materials, and their structures (A and B). Moreover, the confinement potential effect of the first material on the energies is more pronounced when their thickness is large and the core radius is small. So, the external potential effect is more significant when the first shell thickness and potential are small. Also, The binding energy of an on-center (off-center) donor atom is greater (weaker) than that of the exciton, whatever the structure of the confinement potential. In addition, the transition from a type-A to a type-B confinement system has been observed. The findings might be used to modify the electronic and excitonic properties in nanomaterials science.

## Introduction

In recent years, improved material growth techniques have enabled the practical realization of semiconductor-based nanoscopic structures such as quantum wells (QWs), quantum dots (QDs), and quantum rings (QRs). Which have been the most studied, both from a theoretical and experimental point of view, because of the numerous possibilities of application in electronic and optoelectronic devices^1–8^. The QDs are considered as carrier systems of almost zero dimension, since the motion of charge carriers in these structures is limited to well-defined energy values. The interest in these particular nanostructures lies in their novel and exceptional electronic, magnetic, and optical properties^9–11^.

In addition, the advances in materials science have enabled the production of a new generation of heterostructures called core/shell quantum dots (CSQDs) and multi-layer quantum dots with different geometrical shapes and material compositions^12–15^. The interest of these nanostructures leads to control the physical properties of the CSQDs by varying their size/thickness, and leads to adjust their energy levels, as well as the intra- or inter-band transitions by absorption or emission. In the published literature, a lot of researches have been interested in core/shell nanostructures^16–18^. Whenever the shell thickness was raised from 10 nm to 17 nm, the interband transition energies marginally dropped from 2.061 eV to 2.007 eV, according to X-ray diffraction patterns, high-resolution transmission electron microscopy scans, and energy dispersive spectroscopy characteristics^19^. The photovoltaic characteristics of self-assembled core-shell GaN/InGaN wires fabricated on sapphire substrates using metal-organic vapor phase epitaxy have been studied^20^. They have used photocurrent spectroscopy to show that the response assigned to the absorption of InGaN/GaN quantum wells can be seen at wavelengths shorter than 440 nm, and that its contact optimization produces an increase of conversion efficiency with 0.33% and a replenishment factor of 83% under 1 sun (AM1.5G) on single wires. Also, the effect of the first and second shell in InAs$_{x}$P$_{1-x}$/InP/ZnSe multi-layered QDs have been investigated^21^. A red-shift and the enhancement of the quantum yield are caused by the first shell of InP. The ZnSe shell allows to improve the stability of the dots for watery applications, such as near-infrared biomedical fluorescence imaging. In an experience with mapping of the sentinel lymph node, these near-infrared emitting multi-layered QDs were effectively utilized. Among the important theoretical researches, we can cite the study of the electron states and their Raman scattering in core/shell quantum well wire^22^. The authors demonstrated that the core/shell and step-quantum well wires^23^ are highly comparable systems, with the step-quantum well wire’s Raman net gain being substantially bigger than the core/shell quantum well wire’s. In addition, in Ref.^24^ they have demonstrated that the quantum well step-barriers are more effective models than the step-quantum well, though it is less practical than the asymmetric double QW.

There have been several research and proposals on electronic properties in the presence of the donor atom or in interaction with hole (exciton) under the external perturbations such as the hydrostatic pressure, temperature, electric, and magnetic field^25–29^. Furthermore, it should be mentioned that determining electron characteristics (energy and wave function) in the presence of the donor atom is a challenging operation that necessitates theoretical effort. Analytical answers are quite tough to come by in this situation. To solve this challenge, multiple approaches will be used to calculate the electronic state, such as the variational method, DFT calculations, perturbation theory, diagonalization approach, finite difference method, and finite element method, etc. The proposed variational approach is much faster than the direct approach in solving the three-dimensional Schrödinger equation, does not require any special software and produces fairly accurate values of the carrier ground state energy (an error of no more than 2 % of the potential well depth). This method is shown to be an efficient tool to accurately calculate the energy spectrum of different quantum dot models. It also verifies the results obtained so far by various analytical and numerical methods that are currently used in a wide range of quantum dot theoretical studies^32^. Also, neutral excitation density-functional theory is a generalization of restricted density functional theory that maintains all of the benefits of a variational approach while removing any assumptions regarding the spatial confinement of electrons and holes^33^. Overall, this technique provides easy access to optical properties and quasiparticles binding energies at computing costs and scales that are equivalent to those of conventional density functional theory. Within the compact density matrix formalism approximation, the optical absorption coefficients of core/shell/shell quantum dot (CSSQD) are investigated with the presence of a single donor atom^32^. The study discovered a considerable blue shift of the transition energies in the presence of an electric field and a QD size influence, allowing them to be adjusted. Taking into consideration rectangular potential profiles, the magnetic field influence and the presence of the shallow-donor impurity on electron states in a Al$_{0.3}$Ga$_{0.7}$As/GaAs/Al$_{0.3}$Ga$_{0.7}$ multi-layered QD have been investigated^33^. They demonstrated that the photoionization cross-section and transition energies are substantially influenced by magnetic field, QD size, and impurity location. The electronic properties in two different structures would be affected by the disposition of elements with different energy gaps. Versus core radius of CdSe/ZnS/CdSe and ZnS/CdSe/ZnS QDs, the electronic parameters such as the radial probability density and the binding energy have been computed^34^. In laboratory techniques, the binding energy and electron probability density are critical considerations in designing and fabricating QDs with excellent electrical characteristics.

Concerning the exciton behavior, the hole created by an electron when it is brought from the valence band to the conduction band interacts with it, generating an electron-hole pair. Most usually referred to as an exciton. The confined energies and the exciton binding energy are affected by the ratio of CSQD radii, which explains that the radiative recombination lifetime and oscillator strength are very sensitive to geometrical confinement^35^. When the ratio equal 1, the radiative lifetime of the InP/ZnS QD approaches saturation, and their radiative lifetime increases up to 9 ns. The $\vec{k}\cdot \vec{p}$ approach under the Hartree approximation is used to investigate non-linear behavior of the binding energy of exciton produced in spherical QDs with a central core and several concentric layers^36^. In fact, the previous works have studied nanostructures with different geometries but what is more significant in cylindrical than spherical quantum dots is described that the variation of the size of the spherical nanostructures as a function of the geometrical parameter (radius) gives only the behavior of the bulk and quantum dot, on the other hand the variation of the size of the cylindrical nanostructures as a function of the geometrical parameters (radius and the height) gives the results of the bulk to the quantum dot through quantum wells and quantum well wires. In an infinite confinement potential, the electron has a zero probability of being outside the system, this situation is different in the case of a realistic finite potential where the wave function of the electron does not disappear. A better system representation should be obtained when the confining potential’s depth is finite, especially for small cylindrical quantum dot sizes. Then, the cylindrical CSSQD configuration with finite confinement potential becomes more used in commercial devices and their advantage leads to control the energy spectrum with three geometrical parameters: the first and the second radius of the cross section and the height of the cylinder, as well as the material parameter such as the doped material concentration, which allows to tune the stability and the photoluminescence efficiency of the quantum system. In a series of papers published recently^37–39^, with the use of the variational approach, we investigated a number of issues related to the properties of exciton in cylindrical QDs with a finite potential confinement, such as binding energy, interband emission energy, diamagnetic susceptibility, and the Stark shift in the presence of LO phonon and under the influence of hydrostatic pressure, temperature, and electric field. Our analytical analysis leads us to evaluate the critical values of the dot size corresponding to the spatial confinement of the electron-hole pair, also their adaptation to the confinement potential effect^37–40^. Most of the work consists in studying the confinement of quasiparticles in cylindrical nanostructures with finite confinement potential of the first shell and infinite confinement potential of second shell^41–43^. Their numerical calculation shown that the binding energy and optical properties in nanostructures are size-dependent (size of core and shell). The quasiparticles (trion and biexciton) remain stable as the dielectric shell thickness increases in CdSe/ZnS nanowires, however they get unstable in ZnO/ZnMgO nanowires when the dielectric shell thickness surpasses 2.5 nm and 2 nm, respectively^41^. The core size for a fixed shell radius in elliptic cylindrical QDs has a significant influence on the position of photoionization and peak intensity cross section^42^. With the second shell dielectric, the real and imaginary parts peaks of the dielectric function might undergo a redshift or a blue shift^43^.

On the other hand, the effect of second shell in QD nanostructures play a key role in tailoring quantum devices. Until now, there are no known theoretical or experimental reports about the confinement of exciton or donor atom confined in a two-shell cylindrical QD with finite confinement potential of second shell. Considering the possible uses of optoelectronic devices that may be obtained from these types of systems, this is a subject that is still open in the study and merits to be researched. In this work, we have investigated the behavior of the electronic energy and the binding energy of quasiparticles in cylindrical core/shell/shell QD that are related to the potential of the first and second shells. The aluminum concentration characterizes the barriers material depth of the first and second shells. We consider the electron, exciton and electron-donor atom confined in cylindrical core/shell/shell QD, assuming finite confinement potential for the first and second shells. The eigenenergy and binding energy of exciton and electron-donor are calculated using the effective mass approximation in parallel with a variational technique. Depending on the electron energy, the effect of QD size and potential depth on ground state electronic and binding energies is investigated. The organization structure of this paper is as follows: in "Theoretical framework" section contains the theoretical framework of a free electron, electron-donor atom, and exciton. In "Results and discussion" section discusses the core/shell/shell QD energies results. We report in "Conclusions" section our conclusions.

## Theoretical framework

**Figure 1:**
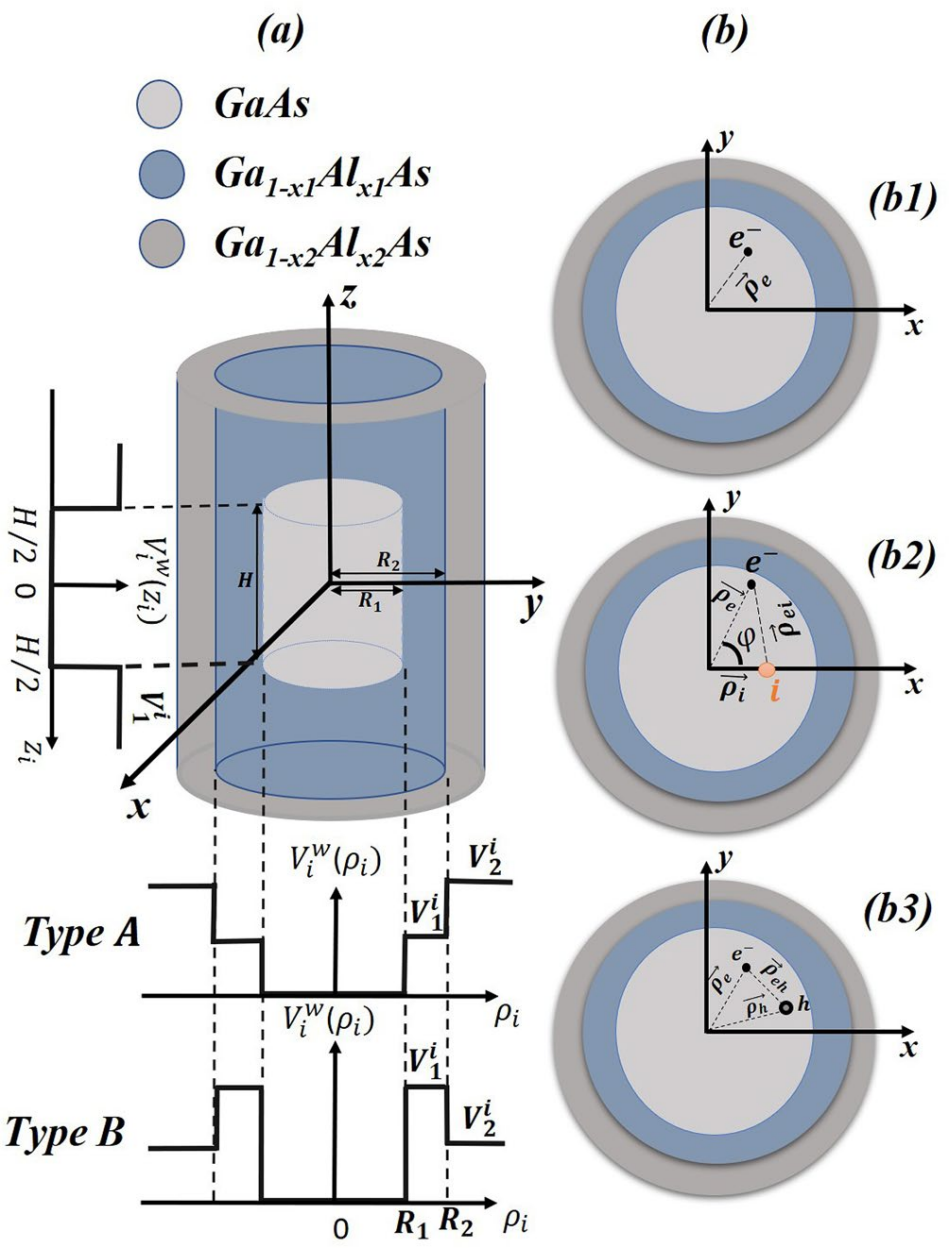
Representation of a cylindrical CSSQD with the geometrical parameters ($R_1$, $R_2$, and *H* are the core, first shell radius, and the core height) and the different type of radial and axial confinements (**a**). In (**b**) is depicted the projection in the *xy*-plane, which illustrates for a single electron in (**b1**), in the presence of the donor atom in (**b2**), and for exciton in (**b3**). Two kind of the confinement potential are depicted (type-A and type-B).

let us consider a GaAs cylindrical QD surrounded by two semiconductors with higher band gap energies. The core material (GaAs) of radius $R_{1}$ and height *H* has a low band gap energy compared to the shells materials, which is illustrated in Fig. 1a. It is covered by first and second shells constituted by Ga$_{1-x1}$Al$_{x1}$As and Ga$_{1-x2}$Al$_{x2}$As, respectively, with the wider band gaps. The first shell thickness is expressed as $R_{12}=R_{2}-R_{1}$, where $R_{2}$ is the first shell radius. The confinement potentials ($V_1$ and $V_2$) considered in our work are finite and controlled by the aluminum concentrations $x_1$ and $x_2$. Two kind of

### Electronic properties

In this section, we consider a single electron confined in cylindrical CSSQD, which prototypical shown in Fig. 1b1, in order to study the quantum and confinements potential effects. Within the effective mass and parabolic band approximation, in the presence of the two confinement potentials, the Hamiltonian for a confined electron is expressed as:

1 $$\begin{aligned} {H_{e}}=-\frac{\hbar ^{2}}{2\,m^{*}_{e(\omega ,b_{1},b_{2})}}\nabla ^{2}_{e}+V^{e}({\rho _{e}},{z_{e})}\,, \end{aligned}$$

where $m^{*}_{e(\omega ,b_{1},b_{2})}$ is the electron effective mass in different regions ($b_{1}$ means the first barrier material Ga$_{1-x1}$Al$_{x1}$As and $b_{2}$ means the second barrier Ga$_{1-x2}$Al$_{x2}$As as well as ${\omega }$ is the QD core). The electron effective mass in the barrier materials as a function of concentration is determined as follow:

2 $$\begin{aligned} m^{*}_{e(b_{1,2})}=m^{*}_{e(\omega )}+0.0835\;x_{1,2}\;m_{0} \end{aligned}$$

where $m^{*}_{e(\omega )}$ is the electron effective mass in the core. The problem’s symmetry allows us to think of the overall confinement $V^{e}({\rho _{e}}, {z_{e}})$ as the sum of the radial and axial potentials. Therefore, the potential may be presented as follows: $V^{e}({\rho _{e}}, {z_{e}})=V^{e}({\rho _{e}})+V^{e}({z_{e}})$, which are expressed by:

3 $$\begin{aligned} {V^{e}({\rho _{e}})}, m^{*}_{e(\omega ,b_{1},b_{2})}= \left\{ \begin{array}{l} 0,\;\; m^{*}_{e(\omega )},\;\;\;\;\; if \;\;\;\;0\le {\rho _{e}} \le {R_{1}}, \\ {V^e_{1}},m^{*}_{e(b_{1})} \;\;\;\; if \;\;\;\; {R_{1}}\le {\rho _{e}} \le {R_{2}},\;\; \\ {V^e_{2}},m^{*}_{e(b_{2})} \;\;\;\; if \;\;\;\; {\rho _{e}}\ge {R_{2}} \\ \end{array}\right. \end{aligned}$$

and

4 $$\begin{aligned} V^e{(z_{e})}, m^{*}_{e(\omega ,b_{1},b_{2})}=\left\{ \begin{array}{l}0, \;\;m^{*}_{e(\omega )}\;\;\;\;\; if \;\;\;\; | z_{e} | \le \frac{H}{2} \\ {V^e_{1}}, \;\;m^{*}_{e(b_{1})}\;\;\;\; otherwise\,. \ \end{array} \right. \end{aligned}$$

The aluminum concentration, $(x_1)$ and $(x_2)$, dependent confinement potential on the barrier materials, $V_1^e(x_1)$ and $V^e_2(x_2)$, respectively, can be expressed in eV as follows:

5 $$\begin{aligned} {V^{e}_{1}(x_1)}=0.658\,(1.115\,x_{1}-0.37\,x^{2}_{1})\, \end{aligned}$$

and

6 $$\begin{aligned} {V^{e}_{2}(x_2)}=0.658\,(1.115\,x_{2}-0.37\,x^{2}_{2})\,. \end{aligned}$$

By using the trial wave function, which is expressed as $\Psi _e{(r)}={\aleph {({\rho _{e}})}}\,{\chi {({z_{e}})}}\,e^{im\varphi _e}$ (where *m* is an integer quantum number), the effective Hamiltonian will be divided on two operators ($H_{\rho }$ and $H_{z}$) associated to the radial a axial directions, i.e.:

7 $$\begin{aligned} {H_e}=H_{\rho _e}+H_{z_e}\,. \end{aligned}$$

Since the quasi-particle motion can be separated in the *xy*-plane (see Fig. 1b1) and the *z* direction, the two time-independent Schrödinger equations are written as:

8 $$\begin{aligned} \left[ -\frac{\hbar ^{2}}{2\,m^{*}_{e(\omega ,b_{1},b_{2}})} \nabla ^{2}_e+V^{e}(\rho _{e})\right] \aleph {(\rho _{e})}=E_{\rho }\,\aleph {(\rho _{e})} \end{aligned}$$

and

9 $$\begin{aligned} \left[ -\frac{\hbar ^{2}}{2\,m^{*}_{e(\omega ,b_{1},b_{2}})} \nabla ^{2}_e+V^{e}(z_{e})\right] \chi {(z_{e})}=E_{z}\,\chi {(z_{e})}\,, \end{aligned}$$

where $\aleph (\rho _e)$ and $\chi {(z_{e})}$ are, respectively, the radial and axial electron wave functions. With the confined electron energy $E_{\rho , z}\le V_1^e$^22^

10 $$\begin{aligned} {\aleph (\rho _e)}=\left\{ \begin{array}{l}J_{m}{(\theta ^{e}_{mn}\,\frac{\rho _{e}}{R_1})}, \;\; if \;\; \rho _{e}\le {R_{1}}\\ {A_{e}}K_{m}{(k^{e}_{mn}\,\rho _{e})}+{B_{e}}I_{m}{(k^{e}_{mn}\,\rho _{e})}, \;\; if \;\; {R_{1}}\le {\rho _{e}} \le {R_{2}} \\ {C_{e}}K_{m}{(\eta ^{e}_{mn}\,\rho _{e})}, \;\;\;\; if \;\;\;\; {\rho _{e}}\ge {R_{2}} \\ \end{array}\right. \end{aligned}$$

and^37^

11 $$\begin{aligned} \chi {(z_{e})}=\left\{ \begin{array}{l}\cos (\frac{l\,\pi }{H}\,z_{e}), \;\;\;\; if \;\;\;\; | z_{e} | \le \frac{H}{2} \\ D_{e}\exp (-l\,\beta _{e}\,|z_{e}|), \;\;\;\; otherwise\,, \ \end{array} \right. \end{aligned}$$

with $l= 1, 2, ...$ and $m= 0, \pm 1, \pm 2, ...$, due to the cylindrical symmetry. The finite confinement potentials effects on the wave function are clearer on the $n^{th}$ root of ${k^{e}_{mn}}$, ${\eta ^{e}_{mn}}$, and ${\beta _{e}}$, which are expressed as follow:

12 $$\begin{aligned}&{k^{e}_{mn}}=\left[ {{\frac{2\,m^{*}_{e(b_{1})}}{\hbar ^{2}}{V^e_{1}}-\left( {\frac{\theta ^{e}_{mn}}{R}}\right) ^2}}\right] ^{\frac{1}{2}}\,, \end{aligned}$$

13 $$\begin{aligned}&{\eta ^{e}_{mn}}=\left[ {{\frac{2\,m^{*}_{e(b_{2})}}{\hbar ^{2}}{V^e_{2}}-\left( {\frac{\theta ^{e}_{mn}}{R}}\right) ^2}}\right] ^{\frac{1}{2}}\,, \end{aligned}$$

and

14 $$\begin{aligned} {\beta _{e}}=\left[ {{\frac{2m^{*}_{e(b_{1})}}{\hbar ^{2}}{V^e_{1}}-\left( {\frac{\pi }{H}}\right) ^2}}\right] ^{\frac{1}{2}}\,. \end{aligned}$$

$J_{m}$ and $I_{m}$ are, respectively, the first type Bessel and modified functions, whereas $K_{m}$ denotes the modified Bessel function of the second order type in all *m*-order cases. To find the constants ${A_{e}}$, ${B_{e}}$, ${C_{e}}$, and ${D_{e}}$ it is important to apply the boundary conditions at ${\rho _{e}}={R_{1}}$, ${\rho _{e}}={R_{2}}$, and ${|z_{e}|}={\frac{H}{2}}$:

15 $$\begin{aligned}&{A_{e}}=\left[ \frac{I_{m}(k^{e}_{mn}{\rho _{e}})J^{'}_{m}({\frac{\theta ^{e}_{mn}}{R_1}}{\rho _{e}})-I^{'}_{m}(k^{e}_{mn}{\rho _{e}})J_{m}({\frac{\theta ^{e}_{mn}}{R_1}}{\rho _{e}})}{I_{m}(k^{e}_{mn}{\rho _{e}})K^{'}_{m}(k^{e}_{mn}{\rho _{e}})-I^{'}_{m}(k^{e}_{mn}{\rho _{e}})K_{m}(k^{e}_{mn}{\rho _{e}})}\right] _{{\rho _{e}}={R_{1}}}, \end{aligned}$$

16 $$\begin{aligned}&{B_{e}}=\left[ \frac{K_{m}(k^{e}_{mn}{\rho _{e}})J^{'}_{m}({\frac{\theta ^{e}_{mn}}{R_1}}{\rho _{e}})-K^{'}_{m}(k^{e}_{mn}{\rho _{e}})J_{m}({\frac{\theta ^{e}_{mn}}{R_1}}{\rho _{e}})}{I_{m}(k^{e}_{mn}{\rho _{e}})K^{'}_{m}(k^{e}_{mn}{\rho _{e}})-I^{'}_{m}(k^{e}_{mn}{\rho _{e}})K_{m}(k^{e}_{mn}{\rho _{e}})}\right] _{{\rho _{e}}={R_{1}}}, \end{aligned}$$

17 $$\begin{aligned}&{C_{e}}=\left[ \frac{1}{K_{m}(\eta ^{e}_{mn}{\rho _{e}})}\left[ {A_{e}}K_{m}(\eta ^{e}_{mn}{\rho _{e}})+{B_{e}}I_{m}(\eta ^{e}_{mn}{\rho _{e}})\right] \right] _{{\rho _{e}}={R_{2}}}\,, \end{aligned}$$

and

18 $$\begin{aligned} {D_{e}}=\left[ \frac{\cos (\frac{l\pi }{H}z_{e})}{\exp (-l\beta _{e}|z_{e}|)}\right] _{|z_{e}|={H/2}}\,, \end{aligned}$$

where ${\theta ^{e}_{mn}}$ and ${\pi }$ are obtained from the following energetic equations:

19 $$\begin{aligned} {A_{e}}{M^{'}_{e}}-{B_{e}}{N^{'}_{e}}=0 \end{aligned}$$

and

20 $$\begin{aligned} \tan \left( \frac{l\pi }{2}\right) =\frac{\beta _{e}}{\pi } H\,, \end{aligned}$$

with

21 $$\begin{aligned} {M^{'}_{e}}={K^{'}_{m}(\eta ^{e}_{mn}{R_{2}})K_{m}(k_{mn}{R_{2}})-K_{m}(\eta ^{e}_{mn}{R_{2}})K_{m}(k^{'}_{mn}{R_{2}})} \end{aligned}$$

and

22 $$\begin{aligned} {N^{'}_{e}}={K_{m}(\eta ^{e}_{mn}{R_{2}})I^{'}_{m}(k_{mn}{R_{2}})-K^{'}_{m}(\eta ^{e}_{mn}{R_{2}})I_{m}(k_{mn}{R_{2}})}\,. \end{aligned}$$

The eigenvalue time-independent Schrödinger equations (Eqs. (8) and (9)) lead to determine the ground state electronic energy as follows:

23 $$\begin{aligned} E_{e}=-\frac{\hbar ^{2}}{2m^{*}_{e(\omega ,b_{1},b_{2})}}\left[ \left( \frac{\theta ^{e}}{R_{1}}\right) ^2+\left( \frac{\pi }{H}\right) ^2\right] \,. \end{aligned}$$

### Electron-donor atom properties

The objective of this section is to examine the off-center atom donor effect on the ground state electronic properties and their corresponding wave functions in a cylindrical CSSQD like the one shown in Fig. 1b2. Within two finite confinements potentials model, the electron-impurity Hamiltonian is given by:

24 $$\begin{aligned} {H_{e-i}}=-\frac{\hbar ^{2}}{2\,m^{*}_{e(\omega ,b_{1},b_{2})}}\nabla ^{2}_e+V^{e-i}_c({\rho _{e}},{z_{e}})+V^{e}({\rho _{e}},{z_{e}})\,. \end{aligned}$$

The resolution of the Schrödinger equation leads to determine the energy and trial wave function of an electron correlated with an off-center donor atom by this second-order differential equation:

25 $$\begin{aligned} \left[ -\frac{\hbar ^{2}}{2\,m^{*}_{e(\omega ,b_{1},b_{2})}}\nabla ^{2}_e+V^{e-i}_c({\rho _{e}},{z_{e}})+V^{e}({\rho _{e}},{z_{e}})\right] \Psi _D{({\rho _{e}},{z_{e}})}=E_D\,\Psi _D{({\rho _{e}},{z_{e}})}\,, \end{aligned}$$

where $\Psi _D{({\rho _{e}},{z_{e}})}$ and $E_D$ are the trial wave function and the ground state energy of electron with impurity, respectively. In the presence of a single donor atom, the trial wave function of electron $\Psi _D{({\rho _{e}},{z_{e}})}$ is written as follows:

26 $$\begin{aligned} \Psi _D{({\rho _{e}},{z_{e}})}=N_D\,{\aleph {({\rho _{e}})}}{\chi {({z_{e}})}}\exp {[-\alpha \rho _{ei}-\gamma {(z_{e}-z_{i})}^{2}]}\,, \end{aligned}$$

with $\alpha$ and $\gamma$ the variational parameters and $N_D$ the normalization constant. The $\exp {(-\alpha \rho _{ei})}\exp {[-\alpha \rho _{ei}-\gamma {(z_{e}-z_{i})}^{2}]}$ describes the correlation effect between the electron and the impurity. The electron-impurity distance in the *xy*-plane is ${\rho _{ei}}=\sqrt{{\rho _e^2+\rho _{i}}^2-2\rho _e\rho _{i} \cos (\theta )}$, whereas the electron and impurity positions in the *z*-direction are $z_{e}$ and $z_{i}$. Also, ${\aleph {(\rho _e)}}$ and ${\chi {(z_e)}}$ are, respectively, the ground-state solution of the Schrödinger equation in the *xy*-plane (lateral direction) and the *z*-direction (axial direction), which are expressed by:

27 $$\begin{aligned} {\aleph (\rho _e)}=\left\{ \begin{array}{l}J_{0}{(\theta ^{e}\frac{\rho _{e}}{R_1})}, \;\; if \;\; \rho _{e}\le {R_{1}}\\ {A_{e}}K_{0}{(k^{e}\rho _{e})}+{B_{e}}I_{0}{(k^{e}\rho _{e})}, \;\; if \;\; {R_{1}}\le {\rho _{e}} \le {R_{2}} \\ {C_{e}}K_{0}{(\eta ^{e}\rho _{e})}, \;\;\;\; if \;\;\;\; {\rho _{i}}\ge {R_{2}} \\ \end{array}\right. \end{aligned}$$

and

28 $$\begin{aligned} \chi {(z_{e})}=\left\{ \begin{array}{l}\cos (\frac{\pi }{H}z_{e}), \;\;\;\; if \;\;\;\; | z_{e} | \le \frac{H}{2} \\ B_{e}\exp (-k_{e}|z_{e}|), \;\;\;\; otherwise\,, \ \end{array} \right. \end{aligned}$$

where $J_0$, $I_0$, and $K_0$ are, respectively, the first type Bessel and first and second modified Bessel functions of $0^{th}$ order. The Coulomb interaction between the electron and the donor impurity is given by:

29 $$\begin{aligned} V_c^{e-i}({r)}=-\frac{ke^2}{4\,{\pi }\,{\varepsilon }\,{\varepsilon _{0}}\,{r}_{ei}}\,, \end{aligned}$$

where *e* is the electron charge, ${\varepsilon }$ is the GaAs static dielectric constant, ${\varepsilon _{0}}$ is the vacuum permittivity, and ${r}_{ei}=|{\overrightarrow{r}_{e}}-{\overrightarrow{r}_i}|=\sqrt{{(\overrightarrow{\rho }_e-\overrightarrow{\rho }_{i})}^{2}+{(z_e-z_{i})}^{2}}$ is the electron-impurity distance (the impurity and electron coordinates are given by ${r_{i}}{({\overrightarrow{\rho _{i}}},{z_{i}})}$ and ${r_e}{(\overrightarrow{{\rho _e}},{z_e})}$, respectively). In addition, $k=0$ when there is not the impurity and $k=1$ when the impurity has been considered. Also, the radial and axial confinement potentials $V^{e}({\rho _{e}},{z_{e}})$ are presented by the Eqs. (3) and (4). In order to more investigate the correlation between the electron and the donor atom, it is important to study the electron-donor binding energy which is defined by the difference between the electronic energy without impurity and the ground state energy of the donor atom.

30 $$\begin{aligned} {E_{b}}={E_{e}}-{E_{D}}\,. \end{aligned}$$

The donor ground-state energy $E_{D}$ is determined by minimizing the expression of average energy:

31 $$\begin{aligned} E_{D}=\min _{\alpha , \gamma }\frac{\langle \psi _{D}| H_{e-i}|\psi _{D}\rangle }{\langle \psi _{D}|\psi _{D}\rangle }\,. \end{aligned}$$

### Excitonic properties

Concerning the Fig. 1b3, we consider an exciton confined in a GaAs cylindrical multi-layered QD. The structure of the nanosystem is similar to the one described above. Within the effective mass approximation and two band model, the basic Hamiltonian of the bound exciton can be described as follows:

32 $$\begin{aligned} {H_{ex}}=\sum _{i=e,h}\left[ -\frac{\hbar ^{2}}{2\,m^{*}_{i(\omega ,b_{1},b_{2})}}\nabla ^{2}_{i}+V^{i}({\rho _{i}},{z_{i}})\right] -\frac{e^2}{4{\pi }{\varepsilon }{\varepsilon _{0}}|\overrightarrow{r}_{e}-\overrightarrow{r}_{h}|}\,, \end{aligned}$$

where $m^{*}_{e(\omega ,b_{1},b_{2})}$ $(m^{*}_{h(\omega ,b_{1},b_{2})})$ is the effective mass of the electron (hole) in different nanostructure regions. $\overrightarrow{r}_{e}=(\overrightarrow{\rho }_{e},z_{e})$ and $\overrightarrow{r}_{h}=(\overrightarrow{\rho _{h}},z_{h})$ are the spatial coordinates of the quasiparticules (electron and hole) in cylindrical CSSQD. The confinement potentials of the quasiparticules $V^{i}({\rho _{i}},z_{i})$ are given by:

33 $$\begin{aligned} {V^{i}({\rho _{i}})}, m^{*}_{i(\omega ,b_{1},b_{2})}=\left\{ \begin{array}{l}0, m^{*}_{i(\omega )}\;\;\;\;\; if \;\;\;\;0\le {\rho _{i}} \le {R_{1}} \\ {V^{i}_{1}}, m^{*}_{i(b_{1})} \;\;\;\; if \;\;\;\; {R_{1}}\le {\rho _{i}} \le {R_{2}} \\ {V^{i}_{2}}, m^{*}_{i(b_{2})} \;\;\;\; if \;\;\;\; {\rho _{i}}\ge {R_{2}} \\ \end{array}\right. \end{aligned}$$

and

34 $$\begin{aligned} V^i{(z_{i})}, m^{*}_{i(\omega ,b_{1},b_{2})}=\left\{ \begin{array}{l}0, m^{*}_{i(\omega )} \;\;\;\; if \;\;\;\; | z_{i} | \le \frac{H}{2} \\ {V^i_{1}}, m^{*}_{i(b_{1})} \;\;\;\; otherwise\, \ \end{array}. \right. \end{aligned}$$

The electron effective mass in the barrier materials is expressed in Eq. (2), as well as the hole effective mass in the barrier materials is given by:

35 $$\begin{aligned} m^{*}_{h(b_{1}, b_{2})}=m^{*}_{h(\omega )}+0.049\;x_{1, 2}\;m_{0} \end{aligned}$$

For the conduction band, the confinement potentials of first and second shells of cylindrical CSSQD are expressed in Eqs. (5) and (6). For the valence band, the confinement potentials expressions are given by:

36 $$\begin{aligned} V_1^h(x_1)= 0.342\,(1.155\,x_{1}+0.37\,x^{2}_{1}) \end{aligned}$$

and

37 $$\begin{aligned} V_2^h(x_2)= 0.342\,(1.155\,x_{2}+0.37\,x^{2}_{2})\,. \end{aligned}$$

Within the reduced atomic units, the energy can be presented by the effective excitonic Rydberg ${R^{*}_{ex}}=\frac{\hbar ^{2}}{2{\mu }a^{2}_{ex}}$, where $a_{ex}^{*}=\frac{{\varepsilon }{\hbar }^2}{e^{2}{\mu }}$ is the exciton Bohr radius and ${\frac{1}{\mu }}=\frac{1}{m^{*}_{e(\omega ,b_{1},b_{2})}}+\frac{1}{m^{*}_{h(\omega ,b_{1},b_{2})}}$ represents the electron-hole reduced mass. The effective Hamiltonian of exciton in cylindrical CSSQD can be expressed as follows:

38 $$\begin{aligned} H_{ex}= & {} -\frac{1}{1+\sigma }\Delta _{e}-\frac{\sigma }{1+\sigma }\Delta _{h}+V^{e}_{\omega }({\rho _{e}},{z_{e}})+V^{h}_{\omega }({{\rho _{h}},{z_{h}}})\nonumber \\ {}- & {} \frac{2}{\sqrt{\rho ^{2}_{eh}+{(z_{e}-z_{h})}^{2}}}\,, \end{aligned}$$

where ${{\sigma }={\frac{{m^{*}_{e(\omega ,b_{1},b_{2})}}}{{m^{*}_{h(\omega ,b_{1},b_{2})}}}}}$. The electron and hole Laplacian operators in the Hylleraas coordinates are written as follows^44^:

39 $$\begin{aligned} \Delta _{e}&=\frac{\partial ^{2}}{\partial \rho ^{2}_{e}} +\frac{1}{\rho _{e}}\frac{\partial }{\partial \rho _{e}}+\frac{\rho ^{2}_{eh} +\rho ^{2}_{e}-\rho ^{2}_{h}}{\rho _{e}\rho _{eh}}\frac{\partial ^{2}}{\partial \rho _{e}\partial \rho _{eh}}\nonumber \\&\quad +\frac{\partial ^{2}}{\partial \rho ^{2}_{eh}}+\frac{1}{\rho _{eh}}\frac{\partial }{\partial \rho _{eh}}+\frac{\partial ^{2}}{\partial z^{2}_{e}} \end{aligned}$$

and

40 $$\begin{aligned} \Delta _{h}&=\frac{\partial ^{2}}{\partial \rho ^{2}_{h}} +\frac{1}{\rho _{h}}\frac{\partial }{\partial \rho _{h}}+\frac{\rho ^{2}_{eh} +\rho ^{2}_{h}-\rho ^{2}_{e}}{\rho _{h}\rho _{eh}}\frac{\partial ^{2}}{\partial \rho _{h}\partial \rho _{eh}}\nonumber \\&\quad +\frac{\partial ^{2}}{\partial \rho ^{2}_{eh}}+\frac{1}{\rho _{eh}}\frac{\partial }{\partial \rho _{eh}}+\frac{\partial ^{2}}{\partial z^{2}_{h}}\,. \end{aligned}$$

In order to calculate the ground state exciton binding energy, the Schrödinger equation $H_{ex}\Psi _{ex}{(\rho _{e},z_e)}=E_{ex}\Psi _{ex}{(\rho _{e},z_e)}$ must be resolved numerically using a variational method. The excitonic envelope wave function is proposed as follows:

41 $$\begin{aligned} \Psi _{ex}{( {r}_{e},{r}_{h})}=N_{ex}\,\Phi _{e}{({r}_{e})}\,\Phi _{h}{({r}_{h})}\,\Phi _{eh}{(\rho _{eh},| z_{e}-z_{h}|)}, \end{aligned}$$

where the wave function term that described the Coulombic interaction is expressed as:

42 $$\begin{aligned} \Phi _{eh}{(\rho _{eh},| z_{e}-z_{h}|)}= \exp {[-\alpha \rho _{eh}-\gamma {(z_{e}-z_{h})}^{2}]} \end{aligned}$$

and

43 $$\begin{aligned} \Phi _{i}{({r_{i}})}=\aleph _{i}{(\rho _{i})}\,\chi _{i}{(z_{i})},\;\;\;(i=e,h), \end{aligned}$$

where $\alpha$ and $\gamma$ are the variational parameters and $N_{ex}$ the normalization constant. According to our system cylindrical CSSQD, the ground state radial and axial wave function of electron and hole would be:

44 $$\begin{aligned} {\aleph (\rho _i)}=\left\{ \begin{array}{l}J_{0}{(\theta ^{i}\frac{\rho _{i}}{R_1})}, \;\; if \;\; \rho _{i}\le {R_{1}}\\ {A_{i}}K_{0}{(k^{i}\rho _{i})}+{B_{i}}I_{0}{(k^{i}\rho _{i})}, \;\; if \;\; {R_{1}}\le {\rho _{i}} \le {R_{2}} \\ {C_{i}}K_{0}{(\eta ^{i}\rho _{i})}, \;\;\;\; if \;\;\;\; {\rho _{i}}\ge {R_{2}} \\ \end{array}\right. \end{aligned}$$

and

45 $$\begin{aligned} \chi {(z_{i})}=\left\{ \begin{array}{l}\cos (\frac{\pi }{H}z_{i}), \;\;\;\; if \;\;\;\; | z_{i} | \le \frac{H}{2} \\ B_{i}\exp (-k_{i}|z_{i}|), \;\;\;\; otherwise\,. \ \end{array} \right. \end{aligned}$$

The exciton ground state binding energy of our system is given by:

46 $$\begin{aligned} E_{B}=E_{e}+E_{h}-E_{ex}\,, \end{aligned}$$

where $E_e$ and $E_h$ are the free electron and hole energies:

47 $$\begin{aligned}&E_{e}+E_{h}=\frac{\hbar ^{2}}{2m^{*}_{e(\omega ,b_{1},b_{2})}}\left[ \left( \frac{\theta ^{e}}{R_{1}}\right) ^2+\left( \frac{\pi }{H}\right) ^2\right] \nonumber \\&\quad +\frac{\hbar ^{2}}{2m^{*}_{h(\omega ,b_{1},b_{2})}}\left[ \left( \frac{\theta ^{h}}{R_{1}}\right) ^2+\left( \frac{\pi }{H}\right) ^2\right] \end{aligned}$$

The exciton ground-state energy, $E_{ex}$, is determined by minimizing the average of excitonic Hamiltonian :

48 $$\begin{aligned} E_{ex}=\min _{\alpha ,\gamma }\frac{\langle \psi _{ex}| H_{ex}|\psi _{ex}\rangle }{\langle \psi _{ex}|\psi _{ex}\rangle }\,. \end{aligned}$$

## Results and discussion

The numerical results of the first shell thickness and potentials forms effects on a single electron energy, electron-donor atom, and exciton ground state binding energy are discussed. In this work we have considered a GaAs cylindrical CSSQD with two confinement potentials (type-A and type-B), which are presented in Fig. 1a. In our numerical calculations, the used parameters are^37^: $m_{e(\omega )}^{*}=0.067\,m_0$, $m_{h(\omega )}^{*}=0.087\,m_0$, and $\varepsilon _r=12.74$, where $m_0$ is the free electron mass. The effective masses considered in our work are approximately independent to the QD nanostructure size. The concentration values $x_{1,2}=0.2,0.3$, and 0.4 are respectively equivalent to confinement potentials $V^e_{1,2}=162,\,250$, and 343 meV for electron and $V^h_{1,2}=84,\,130$, and 178 meV for the hole. The effective Bohr radius ${a^{*}_{ex}}=18$ nm and the effective Rydberg units ${R^{*}_{ex}}=3.0$ meV should be noted. The numerical findings are presented in meV for the energies, and in nm for the lengths describing the core radius, first shell, and height thickness.

**Figure 2:**
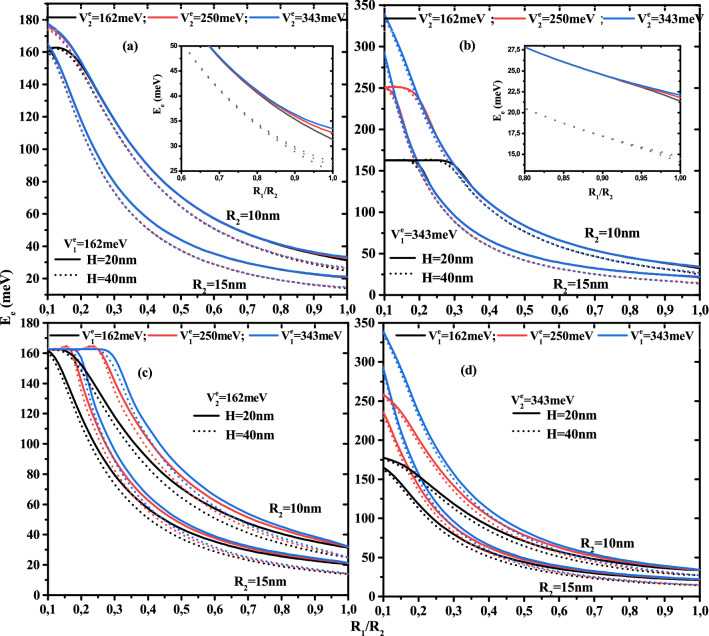
Ground state electronic energy versus the ratio core radius to first shell radius ($R_1$/$R_2$) for different second shell confinement potential with $V^e_1$ = 162 meV (**a**) and $V^e_1$ = 343 meV (**b**) as well as for different first shell confinement potential with $V^e_2$ = 162 meV (**c**) and $V^e_2$ = 343 meV (**d**). Results are for two QD height (H=20 and 40nm) and two first shell radius ($R_2$ = 10 and 15 nm).

The Fig. 2 plots the variation of the electron ground state energy as a function of the core to shell radius ratio $R_1$/$R_2$ for two QD height ($H=20$ and 40nm) and two first shell radius ($R_2$=10 and 15 nm) with two confinement potentials forms: Fig. 2a and d for type-A and Fig. 2b and c for type-B. According to the results of these curves, the electron energy increases when the core, first shell radius, and QD height decrease. The effect of height is more pronounced in the low thickness of the first layer. It is clear that, in the case of type-A confinement potential (see Fig. 2a,d), the electronic energy strongly depends on the potential depth of the first barrier material than on the second barrier material, especially in small core sizes. Similarly, the confinement of the ground state inside the core is clearer in Fig. 2b,c, which shows that in type-B cases, the second potential depth controls their energy. For $V^e_1$= 343 meV, Fig. 2b shows that the electron is confined when the core radius is larger than 2 and 3 nm for the second potential depth equal to $V^e_1$=162 and 250 meV. Furthermore, Fig. 2c shows that, for $V^e_1$= 162 meV, the confinement of the electron ground state increases as the core radius decreases until reaches the potential energy of the first barrier material. This critical values is due to the strong confinement of the electronic wave function inside the core, which is dependent on the confinement potential of the second layer, therefore the increase of the confinement potential of the second barrier material leads to increase the critical value of the core radius such that $R_1$= 1.5, 2, and 3 nm for $V^e_1$= 162, 250, and 343 meV, respectively. The zoom insets in Fig. 2a and b, for a fixed first shell confinement potential ($V^e_1$=162 and 343 meV), show that the second shell confinement potential effect is slightly visible when the core radius $R_1$ is close to the radius of the first shell $R_2$, i.e., the first shell thickness is smaller ($R_{12}\rightarrow 0$). Therefore, the augmentation of the additive confinement ($V^e_2$) leads to augment the electronic energy and it influences is negligible at high confinement core system due to the predominance of the quantum confinement. It is clear that, in Fig. 2c,d, the effect of the first shell confinement is more significant in the small core radius due to the large shell thickness, which produces the strong confinement of the electronic wave function. For a given value of first shell radius and core height, all curves converge to a same electron energy when thickness shell $R_{12}\rightarrow 0$, whatever the structure of confinement potentials.

**Figure 3:**
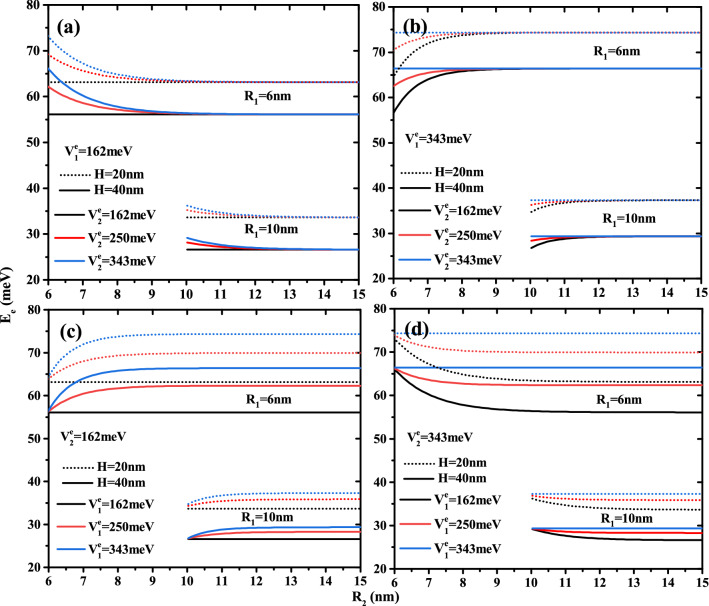
Ground state electronic energy as a function of first shell radius $R_2$ for different second shell confinement potential with $V^e_1$ = 162 meV (**a**) and $V^e_1$ = 343 meV (**b**) as well as for different first shell confinement potential with $V^e_2$ = 162 meV (**c**) and $V^e_2$ = 343 meV (**d**). Results are for two core height ($H=$20 and 40 nm) and two first shell radius ($R_2$ = 10 and 15 nm).

In order to show the impact of the multi-layered on the electronic energy, we have plotted the ground state energy of the electron as a function of the first shell radius $R_2$ for different potential barrier depth $V^e_1$ and $V^e_2$ and different CSSQD size with core radius $R_1$=6 and 15nm and their height $H=$20 and 40 nm. The confinement potentials types, (A) and (B), have been presented, respectively, in Fig. 3a,d and b,c. It is clear that the variation of the electronic energy as a function of $R_2$ remains constant when $V^e_1=V^e_2$. For a given core radius, it is important to note that, in the case of the confinement potential type-A (Fig. 3a,d), the confined electron energy is found to diminishes when the first shell radius $R_2$ is increased until that it saturates. In contrast, in the case of the confinement potential type-B (Fig. 3b,c), the energy increases with the radius $R_2$. This is due to the thickness of the first shell and their energy amount added to the system from the confinement potential $V^e_1$. The strongest confinement zone (low first shell thickness) exhibits a rapid effect of confinement potentials, whereas the weak confinement region exhibits a weak variation of energy growth.

**Figure 4:**
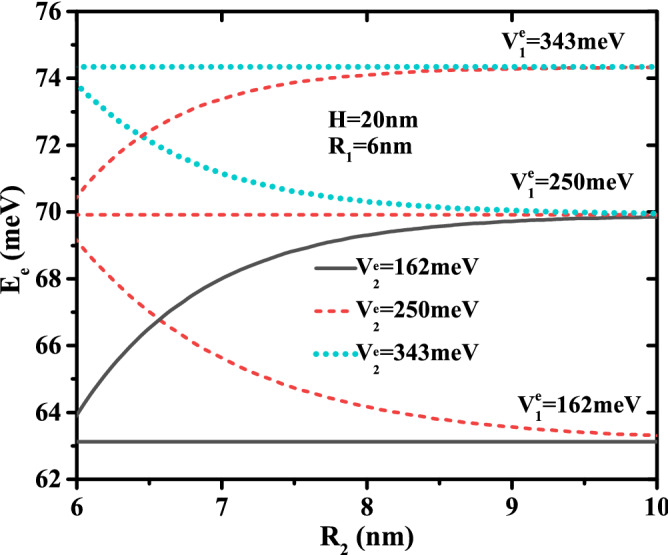
Ground state electronic energy as a function of the first material radius $R_2$ for different confinement potentials ($V{^e_1} = 162,\,250$ and 343 meV) and ($V{^e_2} = 162,\,250$ and 343 meV) with core dimensions $R_1$ = 6 nm and $H = 20$ nm.

The combined effect of two confinement potentials types and layer thickness $R_{12}$ on the energy are presented in Fig. 4, in which the confinement potential effect of the outer layer on the energy decreases with increasing thickness of the first shell and converges to the energy described by their confinement potential.

**Figure 5:**
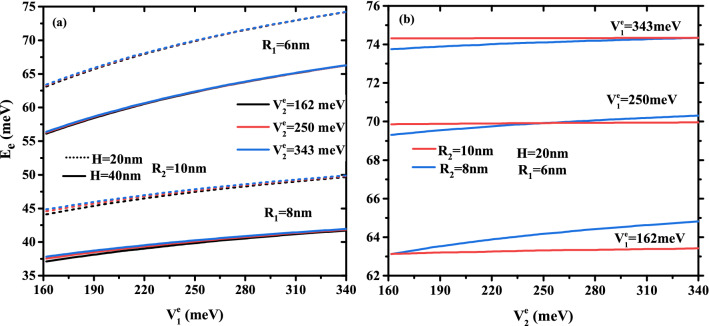
Ground state electronic energy as a function of (**a**) first confinement potential $V^e_{1}$ for different core size ($R_1$ = 6, 8 nm, and $H=$ 20, 40 nm) and three values of external confinement potential depth ($V{^e_2}$ = 162, 250, and 343 meV) with a fixed first shell radius $R_2$ = 10 nm, (**b**) external material confinement potential $V^e_{2}$ for two radius $R_2$ and three confinement potential of first shell material ($V^e_{1}$ = 162, 250, and 343 meV) with a fixed core size ($R_1$ = 6 nm and $H=$ 20 nm).

To see the influence of the multi-layered confinement potentials, the core and first shell radius on the electronic energy, we have plotted in Fig. 5a their behavior as a function of the first layer potential for two CSSQD core radius ($R_{1}$=6 and 8 nm) and three potential of the secondary layer ($V{^e_2}$=162, 250, and 343 meV) with $R_{2}$= 10 nm, and in Fig. 5b as a function of the second layer potential for two shell radius ($R_{2}$=8 and 10 nm) and three potential of the first layer ($V{^e_1}=162,\,250$, and 343*meV*) with $R_{2}=10nm$ and $H=20$ nm. It is seen that the electronic energy increases monotonically with the multi-layered confinement potentials $V_{^e_1}$ and $V_{^e_2}$. However, the confinement potential effect of the outer layer has more influence on the electronic energy when the confinement potential and the thickness of the first layer are smaller. In Fig. 5b, the increase in confinement potential $V^e_2$ is more significant in the case of confinement type-A than type-B, and their effect to change the confinement system from type-A to type-B has been observed at $V^e_1$=252 meV. Thus, their effect on the electronic energy is much more sensitive to the weak first shell thickness and the core radius of the cylindrical CSSQD. It is important to note that the intersection of the electronic energy for the two core radius $R_{2}=8$ and 10 nm is clearer when the potentials $V{^e_1}=V{^e_2}$.

**Figure 6:**
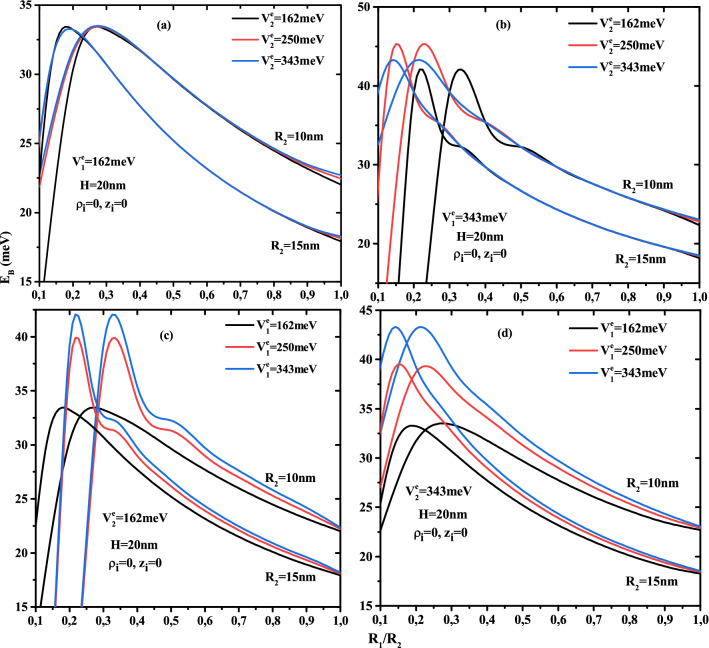
Ground state binding energy of a single donor atom as a function of the core to shell radius ratio $R_1$/$R_2$ for different second shell confinement potential with $V^e_1$= 162 meV (**a**) and $V^e_1$ = 343 meV (**b**), as well as for different first shell confinement potential with $V^e_2$ = 162 meV (**c**), and $V^e_2$ = 343 meV (**d**). Results are for two first shell radius ($R_2$= 10 and 15 nm) and CSSQD height $H=20$ nm.

Due to the Coulomb interaction between the electron and the donor atom, the presence of impurity affects the quantum devices performance as well as their optoelectronic and transport properties. Therefore, we have interested to investigate, in Fig. 6, the electron-impurity binding energy as a function of the core to shell radius ratio for two first layer radius and two confinement potential types (Type-A in (a) and (d), type-B in (b) and (c)) with the a CSSQD height $H=20$ nm. For a given first shell radius and confinement potential with finite barrier, the binding energy increases as the core radius decreases until reaching a maximum value and then decreases. The increase of the binding energy is due to an increase of the Coulombic interaction between the electron and the impurity and it’s decrease with decreasing the core of the CSSQD is produces by the penetration of the electron wave function in the barrier materials by tunneling effect. This effect leads to increases the electron-impurity distance, consequently $E_B$ decreases strongly. It is important to note that, in Fig. 6b–d, the effect of the confinement potential controls on the critical core radius of the tunneling effect. In contrast, according to the results of Fig. 6a, the critical core radius for the electron penetrate into the barrier material equals $R_1$= 2.6 nm, whatever the energy of the confinement potential of the external layer. When the first layer thickness and core radius are smaller, the second shell confinement potential effect is more visible in Fig. 6b, due to the combined effect of the geometrical confinement and the potential energy. Moreover, for the two confinement types (A and B) (in Fig. 6c,d), it is noted that the influence of the confinement potential $V^e_1$ is more significant in the strong confinement region, due to their layer thickness which reduces the overlap of the electron wave function. Furthermore, in Fig. 6b,c, it is clear that the behavior of the binding energy has oscillations when $V^e_1<V^e_2$. This behavior is due in fact when the electron encounters the second potential which is superior to the first one which reinforces the oscillation of the electron to penetrate the second potential barrier.

**Figure 7:**
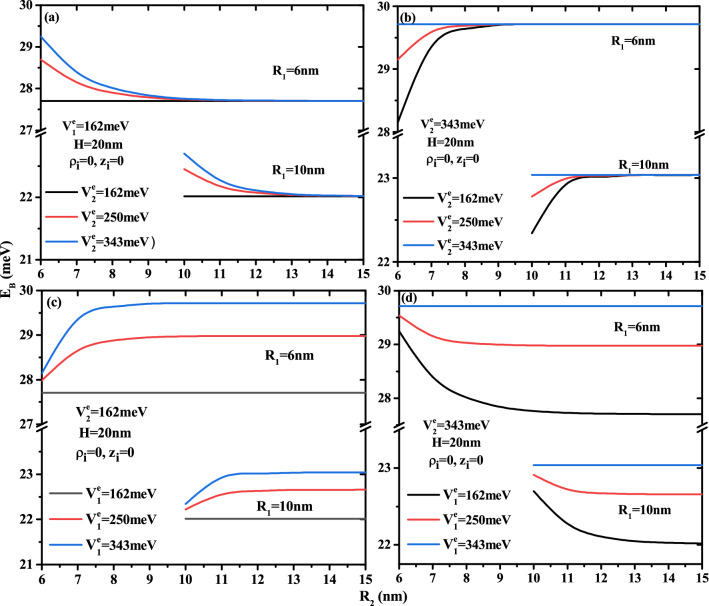
Electron-impurity binding energy as a function of first shell radius $R_2$ for different second shell confinement potential with $V^e_1$=162 meV (**a**) and $V^e_1$ = 343 meV (**b**), as well as for different first shell confinement potential with $V^e_2$ =162 meV (**c**) and $V^e_2$ = 343 meV (d). Results are for two first shell radius ($R_2$ = 10 and 15 nm) and CSSQD height $H=20$ nm.

**Figure 8:**
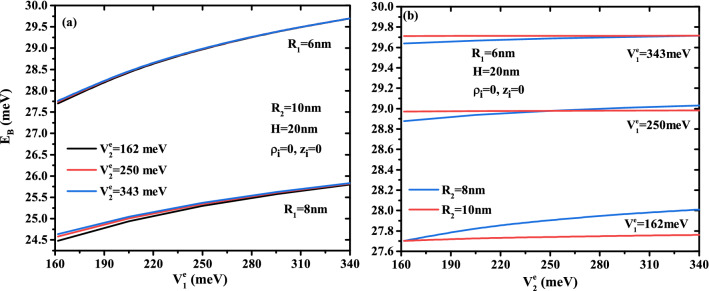
Binding energy of an on-center donor atom versus (**a**) first confinement potential $V^e_{1}$ for different core size ($R_1$ = 6, 8 nm, and $H=20$ nm) and three values of external confinement potential depth ($V{^e_2}$ = 162, 250, and 343 meV) with a fixed first shell radius $R_2$ = 10 nm, (**b**) external material confinement potential $V^e_{2}$ for two radius $R_2$ and three confinement potential $V^e_{1}$ of first shell material with a fixed core size ($R_1$ =6 nm and $H=20$ nm).

The investigation of the donor atom issue in semiconductor QDs is a highly valuable model for comprehending these nanostructures vast features. Because QDs are confined in all 3D, the distance electron-impurity is reduced, resulting in an increase in the Coulomb interaction. it is clear that, in Fig. 7a–d, the binding energy of an on-center donor atom versus the first layer radius $R_2$ keeps the same behavior as in Fig. 3a–d, for different potential barrier depth $V_1$ and $V^e_2$ and different CSSQD size ($R_1$=6, 15 nm, and their height $H=20$ nm). The most important thing is that, in the presence of an on-center impurity, the binding energy in relation to that of the exciton (see below) becomes more important, which produces an increase of the radiative time of the quantum system. Similarly, the variation of the binding energy as a function of the multi-layered confinement potentials $V_1$ and $V^e_2$ presented in Fig. 8a and b, have the same behavior as that seen in Fig. 5, with an energy shift due to the Coulomb interaction.

**Figure 9:**
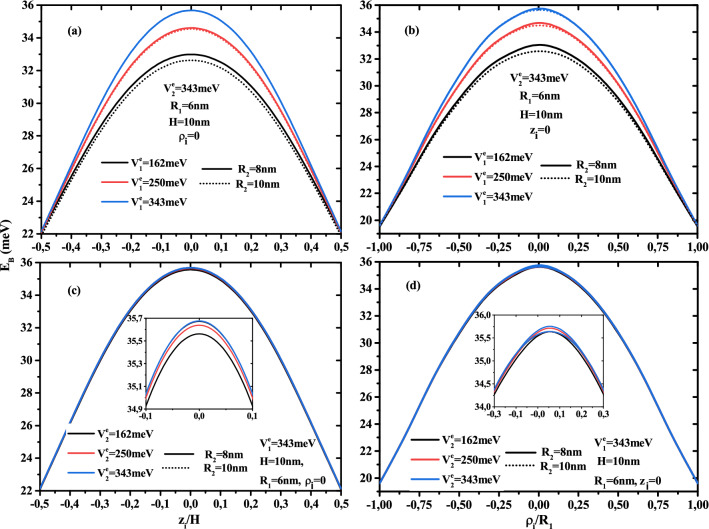
Binding energy of an off-center donor atom as a function of (**a**) the normalized axial impurity position ($z_i/H$), and (**b**) the normalized radial impurity position ($\rho _i/R$) in the type-A confinement potential, (**c**) the normalized axial impurity position ($z_i/H$), and (d) the normalized radial impurity position ($\rho _i/R$) in the type-B confinement potential. Results are with a fixed core size ($R_1$ = 6 nm and $H=10$ nm) for two first shell radius ($R_2$ = 8 and 10 nm).

The donor atom can be placed anywhere on the nanostructures, and its movement in the axial and lateral directions is thought to be a significant role in the binding energy variation. To show the displacement effect along the main directions of cylindrical CSSQD, we have presented in Fig. 9, the ground state binding energy behavior for type-A in Fig. 9a,b and for type-B in Fig. 9c,d. The electron-impurity binding energy dependence on the ratio of the axial (radial) impurity position to the core height $z_i/H$ (radius $\rho _i/R$) is plotted in Fig. 9a,c (Fig. 9b,d). Due to the largest probability density of electron in the core of CSSQD, as shown in Fig. 9a–d, the binding energy reaches its maximum value when the impurity is positioned at the gravity center of the nanostructures. As a result, the electron-donor bound is more stable. When the donor is displaced from the edge to the edge of CSSQD, the binding energy increases until it reaches its maximum value, then it gradually diminish. As a consequence, when the impurity is near to the CSSQD walls, the electron-donor interaction is less coupled and the influence of confinement potentials ($V^e_{1}$ and $V^e_{2}$) is negligible. For this reasoning, we have focused on the donor atom position $z_i=H/2$ and $\rho _i=0$ in the previous figures. For a given shell thickness ($R_{12}$ = 2 and 4 nm), the figures show that the confinement potentials effects ($V^e_{1}$ and $V^e_{2}$) on the binding energy is more significant when the impurity position is near to the center ($z_i=H/2$ and $\rho _i=0$), especially in the case of type-A confinement potential. Furthermore, for a fixed core radius, we can see from the Fig. 9a,b that as the first shell confinement potential (type-A case) augments, the reduction of their thickness leads to enhance the binding energy, and the thickness effect is clearer for $V^e_{1}$ = 162 meV than for 250 and 343 meV, due to the strong confinement of the electronic wave function. Moreover, in Fig. 9c,d, the combined effect of second shell confinement potential and first shell thickness on the binding energy is weaker in the type-B confinement system, this is due to the predominance of the confinement potential of the first layer ($V^e_{1}$ = 343 meV) than the second layer.

**Figure 10:**
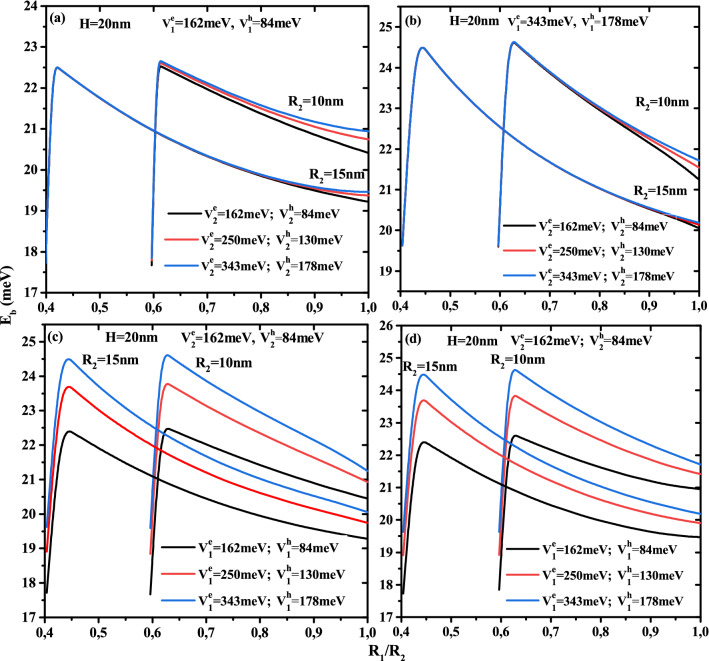
Exciton binding energy as a function of the core to shell radius ratio $R_1/R_2$ for different second shell confinement potential with $V^e_1$ = 162 meV and $V^h_1$ = 84 meV (**a**), $V^e_1$= 343 meV and $V^h_1$ = 178 meV (**b**) as well as for different first shell confinement potential with $V^e_2$= 162 meV and $V^h_2$= 84 meV (**c**), $V^e_2$ = 343 meV and $V^h_2$ = 178 meV (**d**). Results are for two first shell radius $R_2$ =10 and 15nm with core height $H=20$ nm.

Studying the binding energy of a single electron-hole pair, i.e. exciton, leads to characterize the optical properties of the nanostructures. In Fig. 10, we have illustrated the behavior of the exciton binding energy as a function of the core to the first shell radius ratio $(R_1)/(R_2)$ of the GaAs/Ga$_{1-x1}$Al$_{x1}$As/Ga$_{1-x2}$Al$_{x2}$As nanostructure for two radius values $R_2$ and two confinement potentials type-A and type-B, which is presented in Fig. 10a,d and b,c, respectively. The appearance of a very well maximum limit for a finite potential model may be seen in these figures. For a given nanostructure confinement potential type and shell radius, when the core radius reduces, the exciton binding energy increases until it reaches a maximum value, at it collapses to the 3D-limit of exciton as the structure’s dimensions decrease to zero. Due to the finite confinement potentials used in our system, by the tunneling effect, the penetration of the excitonic wave function into the barrier materials increases with decreasing core radius of the CSSQDs, resulting in a decrease in their binding energy. Generally, the first particle that feels the existence of the barriers materials is the electron, because the electron effective mass is lower than that of the hole effective mass. From the Fig. 10a,b, we notice that the increase of the external confinement potential $V^{e,h}_{2}$ leads to an increase of the binding energy mainly when the first layer thickness tends to zero $R_1/R_2=1$, and their influence is more remarkable in the low dimensional confinement systems $R_{2}$ = 10 nm than in intermediate confinement systems $R_{2}$ = 15 nm. Furthermore, the binding energy is rather resistant to the impact of second confinement potential in the strong spatial confinement situation, i.e. for a wide first shell thickness. The combined effect of first shell potential and their thickness on the exciton binding energy are illustrated in Fig. 10c,d. For a given first shell thickness, it can be seen that the binding energy enhances with increasing the first shell potential as well as their thickness, specially, when the core radius is smaller, which leads to adjust the optical coefficients such as linear and nonlinear optical rectifications, second-harmonic and third-harmonic generations.

**Figure 11:**
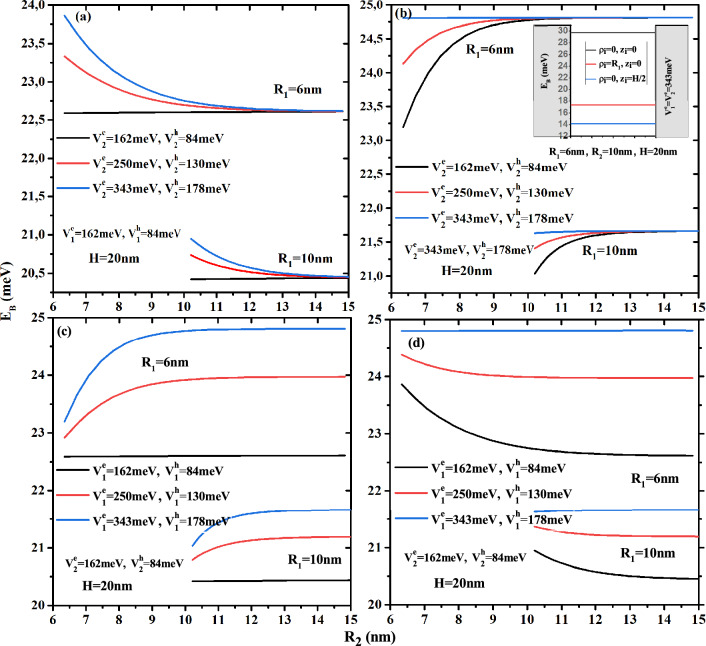
Exciton binding energy as a function of first shell radius $R_2$ for different second shell confinement potential with $V^e_1$ =162 meV and $V^h_1$ = 84 meV (**a**), $V^e_1$ = 343 meV and $V^h_1$ = 178 meV (**b**), as well as for different first shell confinement potential with $V^e_2$ =162 meV and $V^h_2$ = 84 meV (**c**), $V^e_2$ = 343 meV and $V^h_2$ = 178 meV (**d**). Results are for two first shell radius $R_2$ = 10 and 15 nm and CSSQD height $H=20$ nm.

On the other hand, Fig. 11 presents the exciton binding energy as a function of the first layer radius $R_2$ for different potentials barriers depth $V^{e,h}_1$ and $V^{e,h}_2$ and different CSSQD size with core radius $R_1$=6 and 15nm and their height $H=20$ nm. It is clearly seen that the binding energy of exciton versus layer radius $R_2$, for different confinement potential types and core radius $R_1$, keeps the same behavior in the case of electron-impurity, which is presented in Fig. 7. The comparison between Figs. 7 and 11 indicate that, for a fixed CSSQD size and confinement potential, the binding energy of on-center donor atom is more important than of exciton. This is due to the large spatial separation of electron and hole towards the extremes and the fact that small distance $|\overrightarrow{r}_{e}-\overrightarrow{r}_{i}|$ that $|\overrightarrow{r}_{e}-\overrightarrow{r}_{h}|$ in the case that the impurity is located in the center of the dot. On the other hand, in the inset of Fig. 11b the binding energy of the ground state is included for different donor atom position. For a CSSQD characterized by $R_1$= 6 nm, $R_2$= 10 nm, and $H=20$ nm with $V^e_1=V^e_2$= 343 meV, the binding energy of the impurity is equal to 17.3 meV and 14.1 meV when the impurity is located at the radial and axial edge of our nanostructure, which is lower than the binding energy of the exciton $E_B$= 24.8 meV.

**Figure 12:**
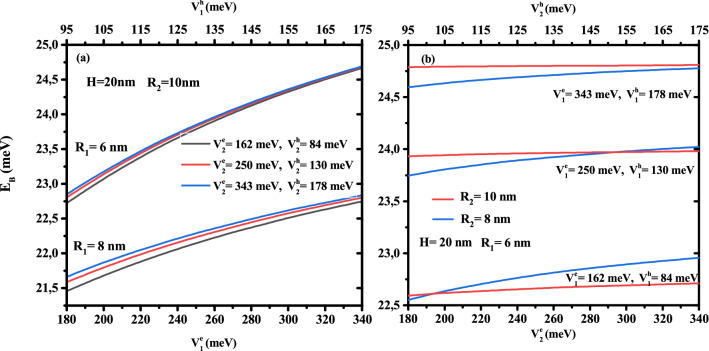
Exciton binding energy versus (**a**) first confinement potential ($V^e_{1}$ and $V^h_{1}$) for different core size ($R_1$ = 6nm, 8 nm, and $H=20$ nm) and three values of external confinement potential depth with a fixed first shell radius $R_2$ = 10 nm, (**b**) external material confinement potential ($V^e_{2}$ and $V^h_{2}$) for two radius $R_2$ and three confinement potential $V^e_{1}$ of first shell material with a fixed core size ($R_1$ = 6 nm and $H=20$ nm).

To better understand the effect of finite multi-layer confinement potentials on the exciton binding energy, we have plotted the exciton ground state binding energy simultaneously as a function of the confinement potential of the electron and hole for the first layer in Fig. 12a and the second layer in Fig. 12b. In Figure 12a, several second potential depths and CSSQD core radius have been considered, whereas in Fig. 12b, three first layer confinement potential depth are combined with their thickness effect; the results are for $H=20$ nm. In Fig. 12a, we can observe that increasing the confinement potential of the first layer ($V^e_{1}$ and $V^h_{1}$) increase the binding energy. For a small values of the first barrier material potential, we notice that the effect of the second barrier material on the binding energy is more significant, and their influences is important when the thickness gets weaker $R_{12}=2$ nm. For a given nanostructure confinement potentials, this figure indicates that the binding energy increases as the core radius decreases, and it is more pronounced for higher first confinement potential. This means that the reduction of the CSSQD leads to reinforce the charge carriers interaction and compressing the exciton wave function in all directions. On the other hand, for a given value of the first confinement potential, Fig. 12b shows that in a large thickness ($R_{12}=4$ nm) the binding energy is not significant when the external confinement potential increases and becomes obvious and more pronounced in small first shell thickness ($R_{12}=2$ nm).

## Conclusions

The present work focuses on the theoretical analysis of the electron, off-center donor atom, and exciton confined in cylindrical CSSQD with finite confinement potentials. The electronic energy, electron-donor, and exciton binding energy are studied in detail by considering the effects of core size, first shell thickness, and shells confinement potentials, $V_1$ and $V_2$. The confinement potential types A and B have been investigated. The numerical resolution of the 3D Schrödinger equation has been made by using the variational method within the effective mass and parabolic band approximations. The results of the theoretical calculations show that the effect of the external layer confinement potential ($V^e_2$) on quasiparticles energies are more significant for the small shell thickness $R_{12}$. With the increase of external potential, the ground state energies of charge carriers increase and their effect becomes negligible when the first shell thickness gets greater. Also, the effect of the first shell confinement is more significant in the small core radius, i.e., in the large shell thickness. For a fixed shell thickness, the electron energy, electron-donor atom, and exciton binding energies increase with the decrease the core radius and the height of CSSQD. Furthermore, the effect to change the confinement system from type-A to type-B has been observed when the potentials $V{^e_1}=V{^e_2}$. The motion of the donor atom in the main axes of the CSSQD has been studied, which shows that their binding energy takes the maximum value when it is localized in the dot gravity center. The confinement potentials effect $V^e_{1}$ and $V^e_{2}$ on the donor binding energy is more significant in the case of type-A confinement potential that in type-B confinement potential when the impurity position is localized near to the center. The comparison between the properties of the donor atom and the exciton indicates that the binding energy of an on-center (off-center) donor atom is greater (lower) than that of the exciton. The findings of numerical modeling revealed that the electronic, with and without impurity, and excitonic features of type-A and type-B CSSQD may be used in further experimental investigation with optimal qualities in laboratory processes.

## Data Availability

The datasets used and/or analysed during the current study available from the corresponding author on reasonable request.
